# Evaluating fuelbreak strategies for compartmentalizing a fire-prone forest landscape in Alberta, Canada

**DOI:** 10.1371/journal.pone.0321722

**Published:** 2025-05-21

**Authors:** Denys Yemshanov, Ning Liu, Eric W. Neilson, Frank H. Koch, Marc-André Parisien

**Affiliations:** 1 Natural Resources Canada, Canadian Forest Service, Great Lakes Forestry Centre, Sault Ste. Marie, Ontario, Canada; 2 Natural Resources Canada, Canadian Forest Service, Northern Forestry Centre, Northwest Edmonton, Alberta, Canada; 3 USDA Forest Service, Southern Research Station, Eastern Forest Environmental Threat Assessment Center, Research Triangle Park, North Carolina, United States of America; Government Degree College Totakan, PAKISTAN

## Abstract

Large wildfires, the dominant natural disturbance type in North American forests, can cause significant damage to human infrastructure. One well-known approach to reduce the threat of wildfires is the strategic removal of forest fuels in linear firebreaks that segment forest landscapes into distinct compartments. However, limited human and financial resources can make it difficult to plan compartmentalization effectively. In this study, we developed a simulation-optimization approach to assist with the planning of wildfire risk mitigation efforts in the Red Rock-Prairie Creek area of Alberta, Canada, a rugged, fire-prone landscape. First, we used a spatial fire growth model to calculate a matrix of fire spread likelihoods between all pairs of locations in the landscape and used this matrix to guide the allocation of firebreaks. Then, we formulated a firebreak compartmentalization problem to reduce the fire spread potential in the landscape. We depicted the landscape as a network of patches containing hazardous fuels and solved a critical edge removal linear programming problem (CERP) to partially fragment the landscape and minimize the potential of wildfires to spread to adjacent areas. We compared the CERP with other fuel treatment strategies intended to minimize fire-threat measures such as burn likelihood and fuel exposure. Compared to these strategies, the CERP solutions demonstrated better capacity to segment the landscape into evenly spaced compartments and effectively minimized fire spread along the prevailing wind paths. Our solutions provide several strategies for reducing the risk of wildfires to forest habitat and could assist strategic planning of wildfire mitigation activities in other regions.

## 1. Introduction

Wildfires are dominant disturbances in the montane and boreal forests of North America [1–4] and have caused significant damage to human wellness, infrastructure and commercial timber supply [5–8]. In Canada, wildfires burn approximately two million hectares of forest annually, with ≈97% of the total area burned by large (10^4^–10^6^ ha) fires [3]. The possible damages and subsequent prevention costs are expected to worsen given that wildfire frequency in North American forests is increasing due to climate change [4,9–13], as exemplified by the record-breaking 2023 fire season [14]. In the United States alone, the total economic burden (costs and losses) associated with wildfires may exceed $348B annually [15].

In forest landscapes, fire management policies, in addition to traditional reactive suppression strategies, institute proactive measures, such as prescribed burns or mechanical treatments, that aim to reduce wildfire hazard by disrupting the spatial connectivity of forest fuels across the landscape [16–19]. Designing and implementing effective fuel treatment systems is difficult and costly in complex landscapes [20–23]. For example, the USDA Forest Service has estimated that adequate reduction of forest fuels, even if limited to high-priority areas, would cost the U.S. $5-6B annually [24,25]. In Canada, hazardous forest fuel treatment strategies are implemented at the national and provincial/territorial level through programs like FireSmart Canada (https://firesmartcanada.ca). Fuel treatments vary substantially among regions of Canada but overall remain relatively sparse compared to other wildfire-prone countries [26].

Development of functional fuel treatment systems can be complicated by uncertain fire weather conditions and insufficient resources that force decision-makers to allocate only a portion of the necessary firebreaks across landscapes [20,23]. Furthermore, the impacts of stand-level fuel reduction measures and other preventive treatments are not always predictable, particularly for landscapes subject to fast-spreading fires or high ignition rates [22,27]. One way to deal with the complexities of firebreak system planning is to segment the forest landscape into compartments delineated by firebreaks that reduce or modify fuel content [17,28–31]. In this setting, the firebreaks are intended to restrict the damage from large fires, if such occur, by containing them within individual partitioned areas.

Wildfire prevention and fuel reduction planning have been aided by optimization models [16,32,33]. For example, Konoshima et al. [34] integrated a fire behavior model with a stochastic dynamic programming model to find an optimal pattern of timber harvest and fuel management under fire risk in a hypothetical landscape. Wei [35], Wei et al. [36] and Wei and Long [37] proposed fuel treatment models to minimize expected losses from wildfires using estimates generated with a stochastic fire prediction model. Chung et al. [38] applied a similar approach to minimize expected losses, incorporating both fire and vegetation growth models into a fuel treatment planning model. Moreover, proposed models have implemented a range of objectives: minimizing burned area in the wildland-urban interface [16], protecting industrial infrastructure and wildlife corridors [39], minimizing fuel treatment costs [40], reducing the probability of high-severity fires [41] and minimize damage in wildland-urban interface areas [42]. Several models have combined fire risk reduction goals with timber harvesting objectives, sometimes by incorporating spatially explicit harvest planning [43–45].

Fuel treatment planning models have been proposed to minimize fuel connectivity across landscapes, including some models focused on firebreak placement [46] and protecting wildlife habitat while reducing fuel connectivity [47]. Optimal planning of contiguous fuel breaks has been addressed with a network flow problem [48] and by solving a critical node detection problem to fragment a fuel network [49,50]. Other proposed approaches included a Stackelberg game model to minimize the impact of the worst-case wildfire outcomes [51], a multi-objective breadth-first prioritization of fuel management within a linear network of fuel breaks in the western U.S. [31] and applications of deep reinforcement learning and metaheuristic approaches to find optimal firebreak designs [52,53].

Firebreak allocation requires an understanding of the behavior of fires not only in terms of their burn probabilities at a particular location, but also where and how far they might spread in a landscape [54]. A common method to approximate potential losses in a wildfire-prone area is to estimate fire intensity and map hazardous fuel patches. Here, a patch refers to a discrete spatial location in a landscape, such as a cluster of forest stands (e.g., depicted as a map cell/pixel). Examples of patch-based measures include the likelihood that fuel is present within a chosen distance from a given patch [55], the estimated burn probability [56–58], the expected burn area for fires ignited in a given patch [48] or large fire probabilities [59,60]. Patch-based measures provide one hazard value per patch (or pixel) and are often presented to decision-makers as maps. While convenient, these measures are insufficient to communicate information about possible fire spread directions, distances, or associated spread probabilities between distinct locations, thereby diminishing their utility to support the planning of fuel reduction measures [22,27,61]. Notably, predicting possible fire spread directions and likelihoods is essential for identifying the possible fire spread corridors in a landscape.

In this study, we compared risk-mitigation fuel reduction strategies based on different performance criteria in a wildfire-prone forest landscape of northwestern Alberta, Canada. We evaluated strategies aimed at reducing fire spread probabilities by minimizing connectivity between patches with hazardous fuels and intersecting major fire spread paths with a network of linear fuel breaks that partition the landscape into compartments. We compared these strategies with a general strategy that aims to reduce area-based fire hazard measures – such as the burn probability or the likelihood of fuel presence within a 500-m distance from a given patch – without considering fuel connectivity or possible fire spread corridors. We solved the fuel connectivity problem with a proposed linear programming model that allocates firebreaks to interdict the potential fire spread corridors in the landscape.

## 2. Methods

### 2.1. Study area

**Fig 1:**
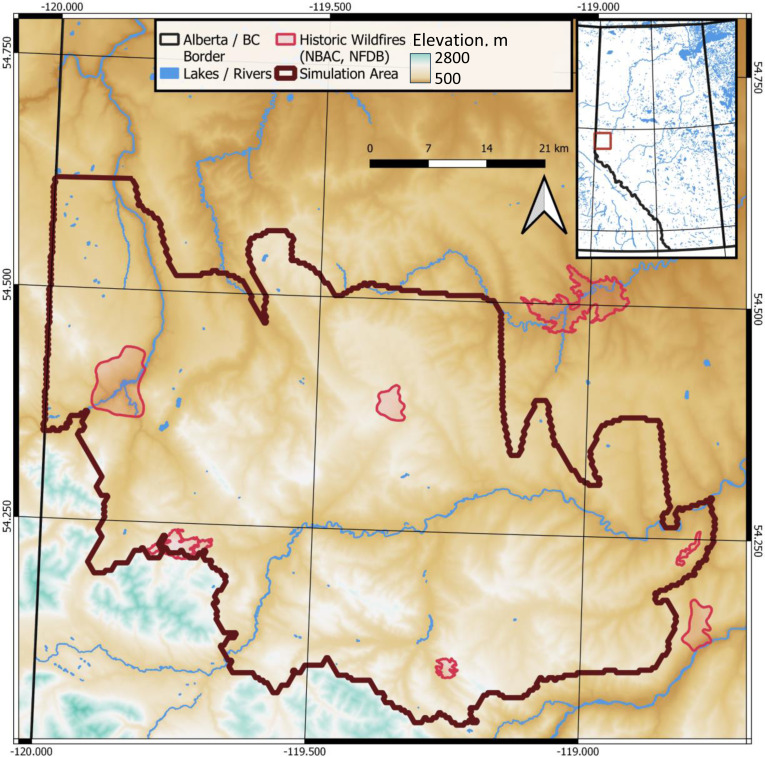
Study area in the Red Rock-Prairie Creek region, Alberta, Canada. Base map data used: administrative boundaries [62]; historic fires [63,64], waterways [65]; digital elevation model [66]. Mapping software used: ESRI ArcMap 10.

We have explored landscape compartmentalization strategies in the Red Rock-Prairie Creek region of northwestern Alberta, Canada (Fig 1).

This landscape includes Rocky Mountains’ upper foothills and lower subalpine ecoregions [67,68]. The area is located within the Peace River basin of Alberta and includes moderate elevations up to 1,750m in the southern portion of the region bordering the Rocky Mountains [68]. The region’s forests are dominated by Engelmann spruce (*Picea engelmannii*) and subalpine fir (*Abies lasiocarpa*) at higher elevations in the subalpine zone, and lodgepole pine (*Pinus contorta*) at lower elevations. Mixedwood stands are composed of aspen (*Populus tremuloides*), balsam poplar (*Populus balsamea*), white spruce (*Picea glauca*), and lodgepole pine (*P. contorta*). The area has been used by forest industry for timber supply [68]. The area has also been subject to oil and gas exploration activities that have left a network of harvested linear features (seismic lines) [69].

**Fig 2:**
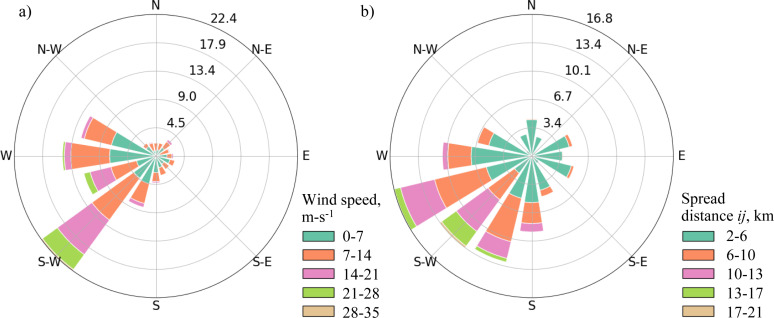
Wind rose diagrams for the study area: a) prevailing winds; b) fire spread indicators *q*_*ij*_ between node pairs *i, j* derived from the fire growth model outputs.

Fire occurrence risk in the study area was estimated as moderate compared to other boreal regions [68]. Winds tend to blow from west-southwest, which influences the dominant fire spread directions (Fig 2a). The historical fire regime was characterized by frequent small to medium-sized fires and rare large fires [68] (Fig 1). Fire return intervals ranged between 80 and 300 years in the subalpine zone in the southern part of the region and between 45 and 476 years in the lower elevation portions of the region [68,70]. Presently, the area has accumulated significant amounts of mature and overmature (100 + years) conifer stands, which have high susceptibility to fires. Approximately 40% of the area was characterized as high to extreme wildfire behavior potential [68]. In the lower-elevation areas in the northeastern part of the region, human-caused fires peak in May, whereas lightning-caused wildfires peak later in the summer. The higher-elevation areas in the southwest experience mostly lightning-caused wildfires, with the peak fire season between May and August [71]. The Government of Alberta has initiated planning efforts to mitigate wildfire risk in the region [72].

### 2.2. Depicting a forest landscape as a fuel network graph

We conceptualized the forest landscape in the study area as a fuel network graph *G* divided into *N* patches *i* with combustible fuels, *i *∈ *N*, (nodes hereafter). A node may include a cluster of multiple forest stands and is considered a firebreak allocation spatial unit. For a pair of adjacent nodes *i* and *j* that are not separated by natural fire barriers, we depicted the fuel connectivity as a set *E* of connecting edges (arcs) *ij* which defined possible vectors of fire spread between adjacent nodes *i* and *j, ij *∈ *E* (see Table 1 for symbolic notations). We chose a hexagonal node grid to minimize the connectivity bias that may be introduced by using irregularly shaped, different-area polygons to delineate forest stands. We assumed the firebreak allocation decisions to be made at the scale of the hexagonal nodes, with the hexagon size chosen to be sufficient for the allocated treatments to reduce the spread of high-intensity fires in the area.

**Table 1:** Summary of the model variables and parameters.

Symbol	Parameter/ variable name	Description
*Sets:*		
*N*	Nodes (forest patches) *i,j,k* in landscape network *G* – potential fuel treatment locations	*i, j, k* ∈ *N*
*E*	Edges connecting adjacent nodes *ij* in landscape network *G*	*E *⊂ *N *× *N*
*N*_*G*_(*i*)	Connected neighborhood which includes node *i*	*N*_*G*_(*i*)∈ *N*
Ω_*i*_	Nodes *j* – members of a fireplain around node *i* (potential spread destinations of fires ignited in *i*)	Ω_*i *_∈ *N*
*Decision variables:*		
*x*_*i*_	Node deletion binary variable (*x*_*i*_ = 0 if node is removed and *x*_*i*_ = 1 otherwise)	*x*_*i*_ ∈{0,1}
*y*_*ij*_	Edge arc deletion binary variable (*y*_*ij*_ = 0 if edge arc *ij* is removed and *y*_*ij*_ = 1 otherwise)	*y*_*ij*_ ∈{0,1}
*u*_*ij*_	Binary variable defining that nodes *i* and *j* are *not* removed and there is a path connecting *i* and *j*	*u*_*ij*_ ∈{0,1}
*S*	Largest fireplain size in landscape network *G*	*S *> 0
*Parameters*		
*p*_*ij*_	Fire spread probability from node *i* to node *j* (based on the fire growth model simulations)	*p*_*ij*_ ∈ [0;1[
*q*_*ij*_	Fire spread binary indicator between nodes *i* and *j*: *q*_*ij* _= 1 for *p*_*ij*_ > 0 and *q*_*ij* _= 0 otherwise	*q*_*ij*_ ∈ {0,1}
*B*	Node (or edge) removal budget limit	*B* > 0
φ_*i*_	Node-based fire hazard measure (burn probability, fuel exposure or the fireplain size, Ω_*i*_)	φ_*i *_> 0
*f*	Scaling factor (small value)	*f* = 0.01
*M*	Large positive (big-M) value	*M* = |*N*|

**Fig 3:**
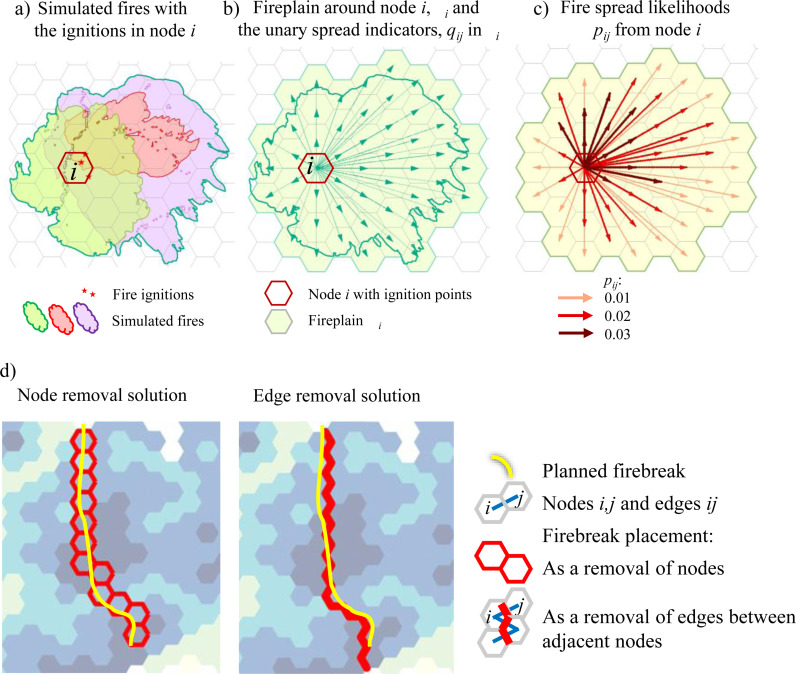
Delineating a fireplain: a) simulated wildfire perimeters with the ignition points in node *i*; b) delineating the fireplain Ω_*i*_ around node *i.* All nodes *j* within a fireplain Ω_*i*_ around *i* are assigned the unary fire spread indicators *q*_*ij*_; c) estimating the fire spread probabilities *p*_*ij*_ from node *i* to nodes *j* from the perimeters of simulated fires (an example of three simulated fires over 100 independent replications of a burn year); d) equivalent removal of nodes vs. edges to allocate a linear firebreak segment.

A fire could potentially spread through pairs of nodes *i* and *j*. The maximum fire spread extent from node *i* to other nodes *j* can be limited by weather, terrain or other fire behavior constraints. For each node *i*, we defined a *fireplain* set of other nodes *j,* Ω_*i*_, to which fires could potentially spread from *i* in realistic circumstances (Fig 3a, 3b). The fireplain concept has been widely used in wildfire science to describe the land area around patch *i* where a wildfire can spread from *i* during a designated timespan [73]. The fireplain configuration and size depend on the connectivity of fuels, terrain and local weather patterns, which may promote or impede the spread of fires. The fireplain set Ω_*i*_ around node *i* is the union of the footprints of all fires which may be ignited in *i* and spread elsewhere (Fig 3a).

Each fireplain Ω_*i*_ around ignition node *i* included the collection of origin-destination node pairs *ij* depicting where a wildfire could spread from *i*. We identified these node pairs with the binary parameter *q*_*ij*_ = 1, *q*_*ij*_ ∈{0,1}, which indicated that fire could spread from *i* to *j* (Fig 3b), and the likelihood values *p*_*ij*_ > 0, *p*_*ij*_ ∈[0;1], that a fire spreads from *i* to *j* (Fig 3c). We used *N* × *N* matrices of *q*_*ij*_ and *p*_*ij*_ values to depict possible fire spread between the nodes in fuel network graph *G*.

We conceptualized firebreak allocation as a partitioning of the fuel network *G* by firebreaks to minimize the number of node pairs *ij* with possible fire spread (i.e., with *q*_*ij*_ = 1 or *p*_*ij*_ > 0). This is consistent with a common strategy for managing fire-prone landscapes by reducing fuel connectivity between forest patches through interdiction of key fire spread paths [47–49,74,75]. We assumed that a pair of nodes *i,j* was connected if network graph *G* contained a path between them and fires could potentially spread from *i* to *j* (so *q*_*ij*_* *= 1 and *p*_*ij *_> 0, and *j* was located in fireplain Ω_*i*_ around *i*).

### 2.3. Modelling the wildfire behavior in a forest landscape

The development of a firebreak system requires knowledge of how wildfires may spread in the landscape. In our setting, we needed to identify all possible ways a wildfire could ignite and spread between nodes in fuel network graph *G*. A common way to depict possible fire behavior in complex landscapes is through application of spatial fire growth models. By simulating stochastic ignition events and subsequent fire propagation from the ignition locations, coupled with the impacts of fuel conditions and weather, these models generate large sets of plausible ignition locations and fire footprints in a landscape as the stochastic realizations of a burn year [60,76–81]. Fire growth models such as Burn-P3 [76] or FSim [77] simulate stochastic ignition events and the fire propagation from the ignition locations [60,78,79] coupled with the impacts of fuel conditions and weather generate multiple fire behavior scenarios [80]. Fire growth models have been developed for many parts of the world, including the Burn-P3 [76], Prometheus [81] and Cell2Fire models for Canada [82] and the FSim model for the U.S. [77]. In this study, we applied the Burn-P3 model [76] to generate stochastic ignitions and fire spread configurations in the study area. The Burn-P3 model uses the Prometheus algorithm [70] to simulate fire growth. For each replication that depicts a fire season, Burn-P3 generates the ignition locations and footprints of individual fires (see details in S1 Appendix).

We generated 60,000 independent simulations of a burn year using the Burn-P3 model, which was sufficient to stabilize the distribution of fireplain sizes. We described fuels using the Fire Behavior Prediction (FBP) System [83]. Burn-P3 modifies fuels based on seasons that determine the presence of deciduous foliage and curing conditions for grass fuels and was parameterized with fuel and terrain data at 100-meter resolution to capture fine-scale fuel heterogeneity. We used the WindNinja software [84] to calculate the effect of local topography on wind directions and wind speeds (see S1 Appendix). To estimate fire weather conditions, we extracted weather data between 2017 and 2019 from Alberta government weather stations located within the study area buffered by a 20-km radius. To simulate the duration of burning of simulated wildfires, we sampled the number of spread days per fire from the historical fire database [85]. The probability of ignitions was stratified spatially based on historical lightning strikes data [86], and the proximity to roads (S1 Appendix).

To act as a strategic fire mitigation measure, the firebreaks needed to be sufficiently wide to ensure their effectiveness against high-intensity fires. High-intensity fires in boreal forests are characterized by the production of firebrands that travel and ignite spot fires [87,88]. To limit the uncertain effect of fire spotting, a sufficient firebreak width would need to be maintained across all allocated segments. We set the hexagon size (the firebreak allocation unit) to 400 ha, which yielded a cross-sectional width of 2.15 km that was sufficient to account for spotting from fires with burn intensities up to 55,000kW/m (based on the spotting distance model from [88]), which accounted for 99.9% of burned locations simulated with the fire growth model.

**Fig 4:**
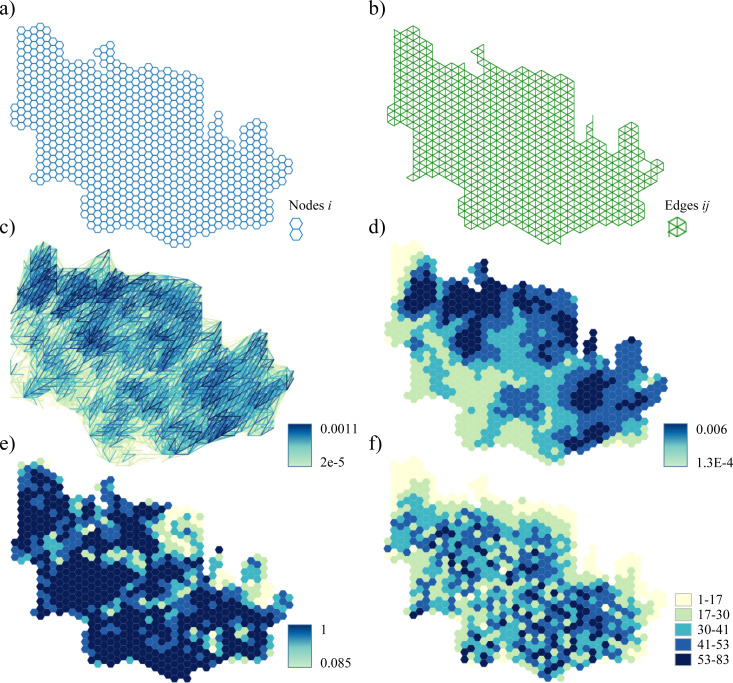
Landscape network *G*: a) nodes *i*; b) edges *ij*; c) fire spread probabilities *p*_*ij*_ between node pairs *i*, *j.* The *p*_*ij*_ values between the adjacent nodes are not shown. Patch-based fire hazard measures, φ_*i*_: d) burn probability; e) the likelihood of fuel presence within a 500-m radius of a given site (rescaled to 0-1 range); f) fireplain size. The network node data can be found in S2 Appendix. Mapping software used: Python Matplotlib 3.4 library.

We applied an approach from our previous work [50] to generate the fireplain sets Ω_*i*_ from the fire growth model outputs. For each node *i*, we assembled all fires from Burn-P3 simulations that were ignited in *i* (Fig 3a) and delineated the fireplain Ω_*i*_ around *i* as a union of the footprints of these simulated fires (Fig 3b). The fireplain set accounts for all fuel discontinuities (e.g., lakes or non-combustible terrain) as well as factors that may have facilitated fire spread from *i*. Destination nodes *j* in fireplain Ω_*i*_, *i *≠ *j,* indicate the locations to which fires ignited in *i* could spread. We estimated the likelihoods of fire spread from *i* to *j*, *p*_*ij*_, as the total number of fires in the fire growth model simulations that were ignited in *i* and able to spread to *j*, divided by the number of fire growth model replications. All node pairs *i*,*j* with *p*_*ij*_ > 0 were assigned the binary indicator values *q*_*ij*_ = 1. The fuel network included 834 nodes and 4788 edges (Fig 4a, 4b). We composed 27,898 binary fire spread indicators *q*_*ij*_ and their corresponding likelihoods *p*_*ij*_ between node pairs *i,j* from the fire growth model outputs (Fig 4c).

### 2.4. Allocating the firebreaks with the network optimization approach

#### 2.4.1. Fire risk mitigation criteria.

We measured the fire spread potential in the fuel break allocation scenarios as the total number of node pairs *i*,*j* between which fire spread was possible (i.e., with *q*_*ij*_ = 1) (Table 2). A similar metric denoted the expected number of node pairs *i*,*j* with fire spread as the sum of the fire spread probabilities *p*_*ij*_ between all node pairs after firebreak placement. To characterize the upper limit of the potential fire spread area, we calculated the maximum size of the fireplain set, *S*, after firebreak placement. Finally, we evaluated three popular patch-based measures which provided single-dimensional fire hazard values for nodes *i*: fireplain size |Ω_*i*_| before firebreak placement, burn probability [57,60] and the probability of fuel presence within a 500-meter radius from a given location [55] (Fig 4d–4f).

**Table 2:** Optimal firebreak models 1-6.

Parameter characterizing fire spread from node *i* to node *j* (or fire hazard)	Objective function (see Table 1 for symbolic notations)	Problem type		
		Critical node detection problem (CNDP)	Critical edge removal problem (CERP)	Node removal to minimize the patch-based fire hazard measure, *φ*_*i*_
Binary fire spread indicator between node pairs *ij*, *q*_*ij*_ Max. fireplain size, *S*	$\min\sum_{i}^{\in N}\sum_{j\neq i}^{j\in \Omega_{i}}u_{ij}q_{ij}$	**Problem 1**	**Problem 3**	
	$\min\left[S+f\sum_{i}^{\in N}\sum_{j\neq i}^{j\in \Omega_{i}}u_{ij}q_{ij}\right]$		**Problem 5**	
Fire spread probability between nodes *i* and *j*, *p*_*ij*_	$\min\sum_{i}^{\in N}\sum_{j\neq i}^{j\in \Omega_{i}}u_{ij}p_{ij}$	**Problem 2**	**Problem 4**	
Patch-based fire hazard measure in node *i, φ*_*i*_	$\begin{array}{c} \min\sum_{i}^{\in N}x_{i}\varphi_{i} \end{array}$			**Problem 6:** *φ*_*I *_= burn probability in site *i* *φ*_*I *_= fuel presence probability within a 500-m distance from a given site *i* *φ*_*I *_= fireplain size before firebreak placement

#### 2.4.2. Problems 1 and 2: Firebreak placement via removal of network nodes.

Our problem 1 (from our previous work [50]) allocated firebreaks in fuel network graph *G* by removal of nodes *i*, which also disrupted the fuel connectivity through those nodes (Table 2). This formulation was based on a critical node detection problem (CNDP), which finds the key nodes in a network whose removal maximally degrades its connectivity according to a chosen criterion, such as the number of location pairs with possible paths between them [50,89–92] or the potential for wildfire transmission to human infrastructure [39].

Firebreak selection in the CNDP was managed by the node removal binary decision variable, *x*_*i*_, which defined whether node *i* was *not deleted* from network *G* (*x*_*i*_ = 1 and *x*_*i*_ = 0 otherwise). For each pair of nodes *i* and *j*, another binary variable, *u*_*ij*_, *i,j* ∈ *N*, indicated that nodes *i* and *j* were *not removed* from network graph *G* (i.e., *u*_*ij*_ = 1 when *x*_*i*_ = *x*_*j*_ = 1) and that there was a fire spread path connecting *i* and *j*, such that, for any pair of adjacent nodes *k* and *l* on that path, *x*_*k*_ = *x*_*l *_= 1. If *u*_*ij*_ = 1, nodes *i* and *j* were located within the same compartment with the possibility of a fire spreading from *i* to *j*, while *u*_*ij*_ = 0 indicated that *i* and *j* were located in separate compartments. Fires could spread between nodes *i* and *j* in both directions with different likelihoods, so node *j* could be a part of a fireplain Ω_*i*_ around node *i,* while *i* might not be a part of a fireplain Ω_*j*_ around *j*. The *u*_*ij*_ and *u*_*ji*_ variables could take different values for node pairs *i,j*, thus helping to incorporate the factors influencing directional spread of fires, such as prevailing winds.

The total number of nodes that could be removed to create the compartments was limited by budget level *B*. Problem 1 objective (1) minimized the number of possible path connections (*u*_*ij*_ = 1) between node pairs in network *G*, which also minimized the fuel network connectivity, i.e.:


$$\min\sum_{i}^{\in N}\sum_{j\neq i}^{j\in \Omega_{i}}u_{ij}q_{ij}$$ (1)

s.t.:


$$\sum_{i}^{\in N}(1-x_{i}leB$$ (2)

$$u_{ij}\geq x_{i}+x_{j}-1\;\forall \text{ (}i,jinE,i\neq j,j\in \Omega_{i}$$ (3)

$$u_{ij}\geq \frac{1}{M}\sum_{k\neq j}^{\begin{array}{c} k\in N_{G}(i),\\ k\in \Omega_{i},j\in \Omega_{k} \end{array}}u_{kj}-(1-x_{i}text\forall \;i\in N,j\in \Omega_{i},\;i\neq j,\text{ (}i,j)\notin E.$$ (4)

The fireplain set Ω_*i*_ limited the scope of decision variable *u*_*ij*_ and equations (1, 3, 4). Constraint (2) defined an upper bound on the number of the removed nodes. Constraint (3) ensured, for an adjacent connected edge between nodes *i* and *j*, that *u*_*ij*_ = 1 if neither node was removed. Constraint (4) ensured that nodes *i* and *j* were connected if there was another non-deleted node *k, k* ≠ *j*, in the connected neighborhood, *N*_*G*_(*i*), that included node *i*, as well as nodes with connections to *i,* such that *k* and *j* were connected. Equation (4) enforced transitive connectivity relationships between nodes in the network graph; for instance, if node *i* was connected to node *j* and *j* was connected to node *k*, then *i* was connected to *k*. The CNDP problem (1–4) was based on an efficient formulation proposed in [91,92], however it was proven to be NP-hard with no polynomial-time approximation [90,93].

In problem 1, all path connection variables *u*_*ij*_ within fireplains Ω_*i*_ were treated as equal irrespective of the fire spread likelihoods between nodes *i* and *j*. Since the number of spread vectors between all node pairs in a landscape compartment decreases (or increases) in quadratic proportion to the linear size of the isolated compartment, fragmenting the landscape into compartments tended to minimize the number of long spread vectors *ij* (which also reduced the spread of large fires).

Our CNDP problem 2 considered scenarios when information about fire behavior was sufficient to estimate the continuous probabilities, *p*_*ij*_, of fire spread between every pair of nodes *i,j*, *p*_*ij*_ ∈ [0;1]. We used the *p*_*ij*_ values as priority weights to rescale decision variable *u*_*ij*_ in the objective function equation (Table 2). The CNDP problem 2 was formulated analogously to problem 1 except the number of node pairs with fire spread paths *ij* in the objective equation was weighted by the likelihoods of fire spread between *i* and *j*, i.e.,:


$$\min\sum_{i}^{\in N}\sum_{j\neq i}^{j\in \Omega_{i}}u_{ij}p_{ij}$$ (5)

s.t.: constraints (2–4).

Objective function (5) minimized the spread of fires with the highest *p*_*ij*_ values. Since the fire size distribution in forest landscapes generally follows a power law [94], objective (5) minimized the spread of the smallest, most frequent fires.

#### 2.4.3. Problems 3 and 4: Landscape partitioning via removal of network edges.

Removing a node in problems 1 and 2 implied the removal of fuels across the entire node area, which also removed all edges connected to that node. In practical conditions, firebreaks and other fuel barriers tend to be established as linear segments with fuel removals at a depth that is sufficient to reduce potential fire intensity enough for a fire to be contained [20,95]. To make the allocation of firebreaks less dependent on the node size, we reformulated models 1 and 2 as a critical edge removal problem (CERP) that disrupts fuel connectivity by removing edges between adjacent nodes (Table 2). Edge removal was controlled by a binary decision variable, *y*_*ij*_, that defined whether edge arc *ij* connecting adjacent nodes *i* and *j* was *not deleted* from network *G* (*y*_*ij*_ = 1 and *y*_*ij*_ = 0 otherwise, *i,j* ∈ *E*). Removing an edge *ij* was analogous to establishing a linear firebreak segment along the border between nodes *i* and *j*. Subsequently, the critical edge removal problem 3 (CERP) was written as follows:

minimize objective (1),

s.t.:


$$u_{ij}\geq y_{ik}+u_{kj}-1\;\forall \;i\in N,k\in N_{G}(i),j\in \Omega_{i},j\in \Omega_{k},i\neq j,$$ (6)

$$u_{ij}\geq y_{ij}\;\forall \text{ (}i,jinE,i\neq j$$ (7)

$$y_{ij}=y_{ji}\;\forall \text{ (}i,jinE,i\neq j$$ (8)

$$\sum_{\begin{array}{c} i,j\in E\\ i<j \end{array}}^{.}(1-y_{ij}leB$$ (9)

$$\sum_{\begin{array}{c} i,k\in E\\ k\neq j \end{array}}^{.}(1-y_{ik})+\sum_{\begin{array}{c} j,l\in E\\ l\neq i \end{array}}^{.}\left(1-y_{jl}\right)\geq 1-y_{ij}\;\forall \text{ (}i,jinE,i\neq j.$$ (10)

Constraint (6) is analogous to constraint (3) and ensured that nodes *i* and *j* were connected (i.e., *u*_*ij*_ = 1) if there was a non-deleted node *k* in the connected neighborhood of *i*, *k* ∈ *N*_G_(*i*), *k* ≠ *j*, such that *k* and *j* were connected. Constraint (7) ensured that adjacent nodes *i* and *j* were connected (i.e., *u*_*ij*_ = 1) if there was a non-deleted edge between *i* and *j,* so *y*_*ij*_ = 1. Constraint (8) specified that the removal of arcs between nodes *i* and *j* occurred in both directions when a firebreak was installed between *i* and *j.* Constraint (9) defined an upper bound *B* on the number of edges that could be removed. Constraint (10) prevented removal of isolated single-edge segments and required that removal of an arc *ij* was accompanied by removal of at least one more arc between node *i* (or node *j*) and another node adjacent to *i* or *j*. Subscript *k* denoted the other nodes adjacent to node *i*, *k* ≠ *j*, and subscript *l* denoted the other nodes adjacent to node *j*, *l* ≠ *i*.

Like CNDP problem 2, the CERP problem 4 applied the continuous fire spread likelihoods *p*_*ij*_ instead of the binary indicators *q*_*ij*_ between node pairs *ij* and minimized the risk of spread of the most frequent fires (Table 2). CERP Problem 4 was formulated as minimizing objective (5), subject to constraints (6–10).

#### 2.4.4. Problem 5: Reducing the largest fireplain area.

Alternatively, managers may seek to reduce the risk of very large fires by limiting the size of the largest potential fire spread area. Limiting the potential fire spread area is analogous to minimizing the size of the largest fireplain Ω_*i*_. Following previous problem formulations minimizing the largest size of isolated components in networks [89,92,96,97], we introduced a non-negative variable *S* that tracked the largest size of fireplains Ω_*i*_ in network *G* and solved the problem by minimizing *S.* In order to reduce the overall fire spread risk while minimizing the largest fireplain size *S*, we added a term to the objective equation that minimized the number of node pairs *i,j* with un-interdicted path connections from objective (1) (Table 2). Our problem 5 minimized the weighted sum of the largest fireplain size *S* and the number of node pairs in network *G* where fire spread was possible, i.e.,:


$$\min\left[S+f\sum_{i}^{\in N}\sum_{j\neq i}^{j\in \Omega_{i}}u_{ij}q_{ij}\right],$$ (11)

s.t.: constraints (6–10) and


$$S\geq \sum_{j\in \Omega_{i}\backslash \{i\}}u_{ij}+1\;\forall \;i\in N.$$ (12)

Symbol *f* defined a relative importance for the fire spread term. The second term in equation (11) helped break ties in optimal solutions. Constraint (12) calculated the value of decision variable *S* as the largest number of node pairs with non-interdicted fire spread paths from node *i* plus 1 (for the node *i* itself), which is the largest fireplain size.

#### 2.4.5. Problem 6: Fuel treatments guided by patch-based fire hazard measures.

For comparison, our problem 6 evaluated a firebreak allocation guided by a patch-based fire hazard measure, *φ*_*i*_, i.e.,:


$$\begin{array}{c} \min\sum_{i}^{\in N}x_{i}\varphi_{i} \end{array}$$ (13)

s.t.: constraint (2).

Problem 6 allocated firebreaks to minimize the sum of the fire hazard measure *φ*_*i*_ in the remaining untreated nodes. We evaluated three popular measures: burn probability, the probability of fuel presence within a 500-m distance and the fireplain size (Table 2).

Since our primary intent was to examine the utility of the CNDP and CERP for partitioning the landscape into compartments on their own, we did not include the constraints enforcing the linear contiguity of the firebreaks. Potentially, other constraints could be added to allocate firebreaks as connected segments [48,50], however this would mask the capacity of problems 1–6 to delineate the compartments on their own and might lead to suboptimal fire hazard reduction.

To compare the node removal and edge removal solutions, we needed to define a relative budget ratio for removing an equivalent number of nodes versus number of edges. The principal aspect for determining this ratio was the number of nodes or edges to delineate an equivalent-length linear firebreak segment (Fig 3d). Through a series of tests, we set the budget ratio of removing a node versus removing an edge as 2.5:1.

### 2.5. Finding the firebreak allocation solutions in the study area

We found the firebreak allocation solutions for a range of budgets between 12 and 120 removed nodes in problems 1, 2 and 6 and, equivalently, between 30 and 300 removed edges in problems 3–5. To determine the trade-offs between the goals of minimizing the number of possible fire spread path connections versus minimizing the fireplain size, we calculated the values of decision variables *S* and *u*_*ij*_ in all problem instances and plotted the solutions in dimensions of *S* and the total number of node pairs in network *G* where fire spread was possible, $\sum_{i}^{\in N}\sum_{j\neq i}^{j\in \Omega_{i}}u_{ij}q_{ij}$.

**Fig 5:**
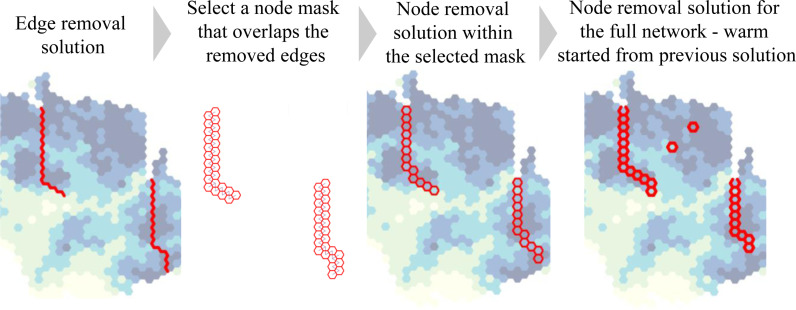
Using the edge removal problem 3 solution to warm start node removal problem 1.

We solved problems 1–6 with the GUROBI linear programming solver [98]. Problems 1 and 2 were smaller in size than problems 3 and 4 but had weaker root relaxations and took 3–5 times longer to solve. We solved problems 1 and 2 for 72 hours or until reaching an optimality gap of 1%, whichever came first. Problems 3 and 4 were solved to optimality within less than 18 hours. To reduce the solving time for the CNDP problems 1 and 2, we used the edge removal problem 3 and 4 solutions to warm start the CNDP problems 1 and 2. First, we selected all nodes with one or more edges removed in problem 3 and 4 solutions as a mask and found the node removal solutions within that mask (assuming the equivalent node removal budgets were 2.5 times smaller than the edge removal budgets) (Fig 5). We then used these solutions to warm-start the full node removal problem. Problem 5 was warm-started from the problem 3 solution.

## 3. Results

### 3.1. Mapping the fire spread patterns

**Fig 6:**
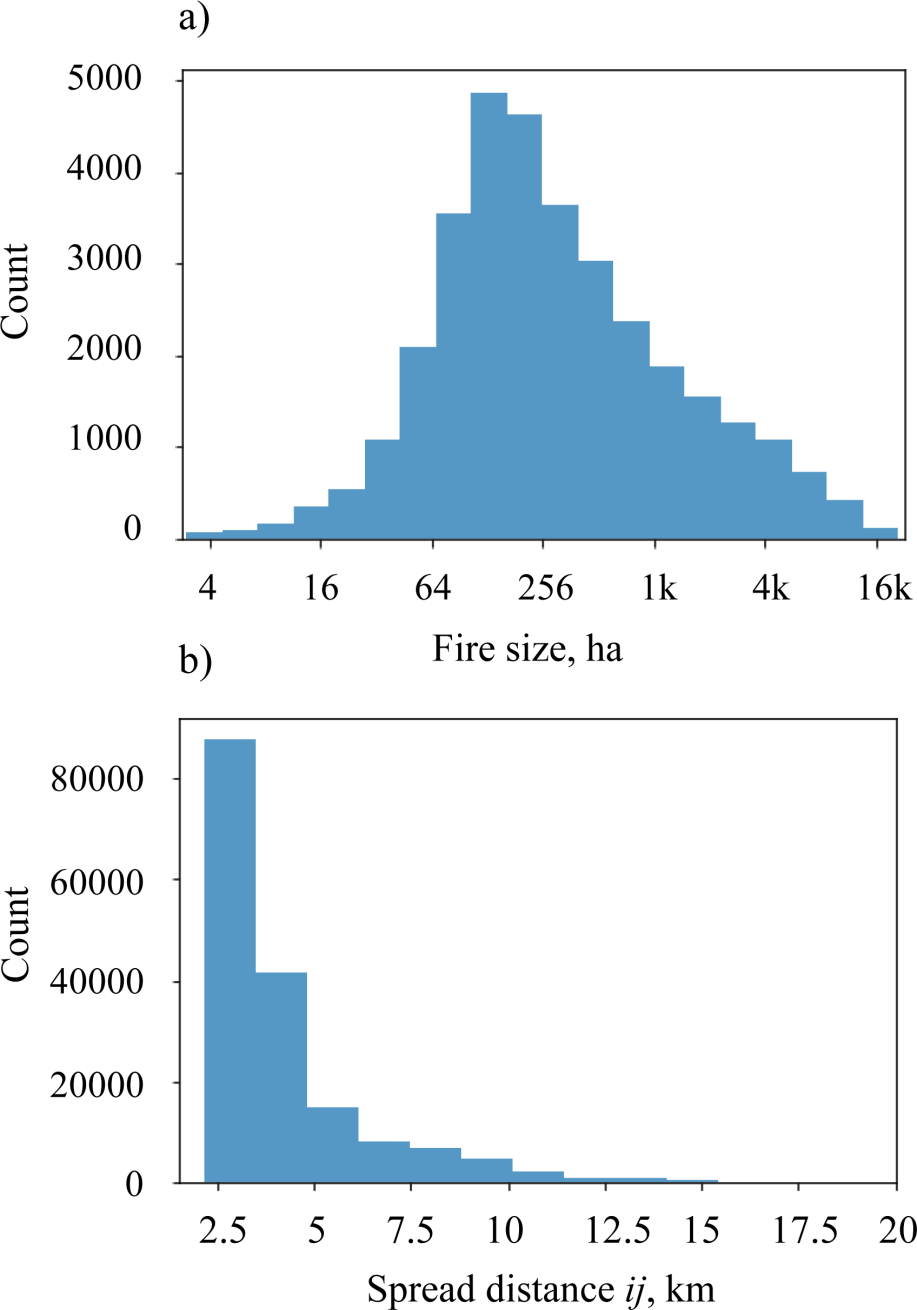
Distributions of the simulated fire sizes and the fire spread vector lengths between node pairs *i, j* in network *G*: a) simulated fire size distribution; b) distribution of fire spread distances between node pairs *i*, *j*, weighted by the estimated fire spread probabilities between *i* and *j*, *p*_*ij*_.

The estimated fire spread patterns in the study area were strongly influenced by the prevailing winds blowing from the west-southwest (Fig 2). The lower-elevation northern and eastern portions of the study area were characterized by higher fire risk, whereas the southwestern portion of the region with higher elevations was characterized by lower fire risk (Fig 4d). The area was characterized by an almost uniform distribution of fireplain sizes (Fig 4f), which indicated that large fires could occur in nearly any part of the study region. The predicted fire size distribution was dominated by small fires, with 95% of fires spreading over distances of 12.9 km or less (Fig 6). The maps of the fire spread likelihoods *p*_*ij*_ between node pairs *i,j* followed the general patterns of burn probabilities (Fig 4c, 4d).

### 3.2. Comparing the firebreak allocation solutions

**Fig 7:**
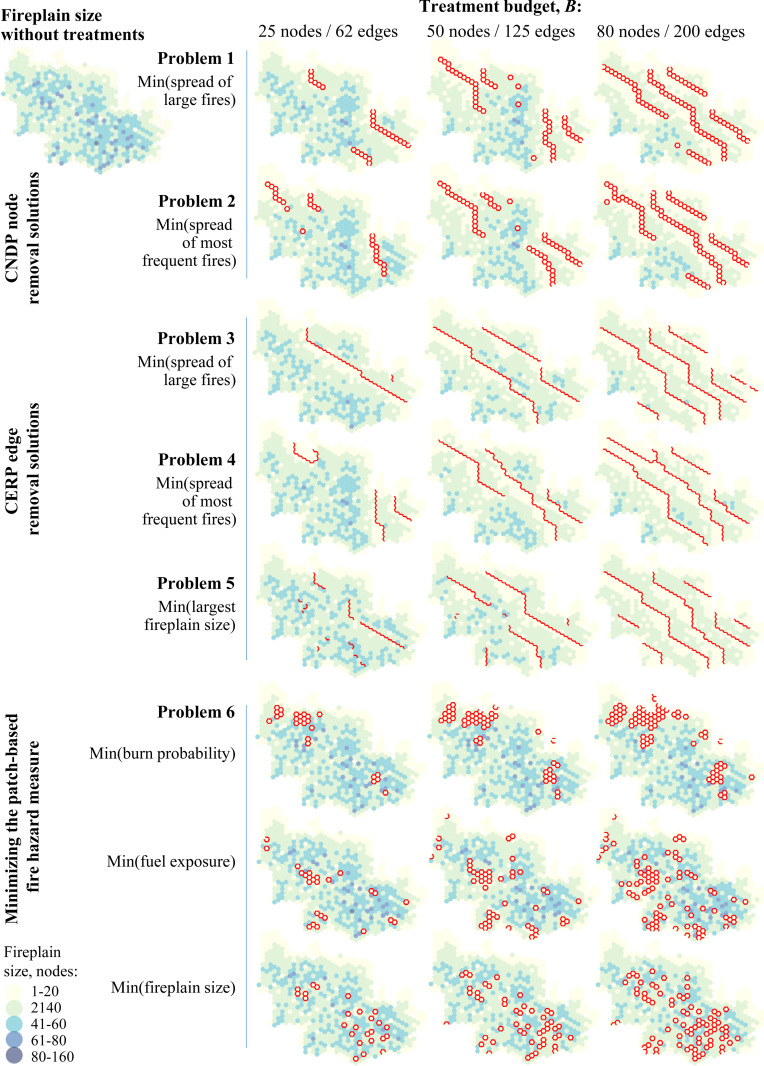
Optimal node and edge removal solutions with treatment budgets of 25, 50 and 80 removed nodes (or equivalently, 62, 125 and 250 removed edges). The cost ratio between the removal of a node in the CNDP solutions and an edge in the CERP solutions is 2.5:1. The background maps show the fireplain size for each node after the treatments. The network node data can be found in S2 Appendix.

We compared the firebreak allocations and the fireplain sizes |Ω_*i*_| after the treatments in Fig 7. The firebreaks tended to be oriented along a northwest–southeast axis – roughly perpendicular to the directions of the prevailing winds (Fig 2a). Almost all removed nodes and edges in the problem 1–4 solutions were allocated in linear segments. In the CNDP problem 1 and 2 solutions, treatments were allocated to a few isolated nodes, but in the CERP problem 3 and 4 solutions all treatments were allocated as linear segments (Fig 7, Table 3). As the treatment budget level increased, problem 3 and 4 solutions fragmented the region into a system of compartments that significantly reduced the largest and mean fireplain sizes (Table 3). Problem 3 solutions, which tended to reduce the spread of the largest fires, revealed even-spaced compartments of similar depth in directions perpendicular to the prevailing winds. Problem 4 solutions, which minimized the spread of the most frequent (small) fires, tended to place more firebreaks in areas with relatively higher burn probabilities (Figs 4d and 7).

**Table 3:** Firebreak allocation summaries in problems 1-6 optimal solutions.

Treatment budget, equivalent edges/nodes	Problem	Relative fire spread risk, vs. no-treatment scenario		Fireplain size, nodes:		Budget portion spent on:		
		Large fires: $\sum_{i}^{\in N}\sum_{j\neq i}^{j\in \Omega_{i}}u_{ij}q_{ij}/\sum_{i}^{\in N}\sum_{j\neq i}^{j\in \Omega_{i}}q_{ij}\;$	Frequent fires: $\sum_{i}^{\in N}\sum_{j\neq i}^{j\in \Omega_{i}}u_{ij}p_{ij}/\sum_{i}^{\in N}\sum_{j\neq i}^{j\in \Omega_{i}}p_{ij}\;$	Max, *S*	Mean	Linear fire breaks	Non-linear clusters >2 nodes	1-node/ ≤ 3-edge segments
–	No treatments	1	1	83	35	–	–	–
	Problem 1 (CNDP)	0.830	0.881	71	30	100%	–	–
	Problem 2 (CNDP)	0.848	0.873	72	30	92%	–	8%
	Problem 3 (CERP)	0.846	0.910	73	29	95.2%	–	4.8%
62 edges	Problem 4 (CERP)	0.857	0.901	73	29	100%	–	–
[25 nodes]	Problem 5 (CERP)	0.912	0.950	54	31	75.8%	–	24.2%
	Problem 6: Min(BP)	0.952	0.924	79	33	–	76%	16%
	Problem 6: Min(FE)	0.959	0.944	81	33	12%	32%	28%
	Problem 6: Min(FS)	0.948	0.938	63	32	40%	–	60%
	Problem 1 (CNDP)	0.711	0.759	68	26	92%	–	8%
	Problem 2 (CNDP)	0.710	0.759	61	26	96%	–	4%
125 edges	Problem 3 (CERP)	0.711	0.826	56	25	100%	–	–
[50 nodes]	Problem 4 (CERP)	0.725	0.814	59	25	100%	–	–
	Problem 5 (CERP)	0.747	0.852	46	26	94.4%	–	5.6%
	Problem 6: Min(BP)	0.917	0.872	79	32	–	86%	6%
	Problem 6: Min(FE)	0.884	0.882	80	32	10%	50%	25%
	Problem 6: Min(FS)	0.870	0.861	57	30	34%	–	28%
	Problem 1 (CNDP)	0.546	0.645	55	21	98.7%	–	1.3%
	Problem 2 (CNDP)	0.559	0.637	56	22	98.7%	–	1.3%
200 edges	Problem 3 (CERP)	0.581	0.730	51	20	100%	–	–
[80 nodes]	Problem 4 (CERP)	0.598	0.721	52	21	100%	–	–
	Problem 5 (CERP)	0.593	0.745	37	21	100%	–	–
	Problem 6: Min(BP)	0.861	0.808	79	31	–	92.5%	5%
	Problem 6: Min(FE)	0.869	0.839	79	31	18.8%	38.8%	28.8%
	Problem 6: Min(FS)	0.813	0.798	53	28	20%	33.8%	23.8%

Problem 6 objective:

Min(BP) – minimize the sum of the burn probability values of the remaining untreated sites in landscape network *G*;

Min(FE) – minimize the sum of the fuel exposure values (the probabilities of fuel presence within a 500-m radius from site *i*) of the remaining untreated sites in landscape network *G*;

Min(BP) – minimize the sum of the fireplain sizes, | Ω_*i*_ | of the remaining untreated sites in landscape network *G*.

The problem 5 solutions, which minimized the largest fireplain size, also generally followed a strategy of fragmenting the landscape into even-spaced compartments but allocated a portion of treatments to block some nodes with large fireplains (Fig 7, Table 3). Given that the threat of large fire occurrence is almost omnipresent across the study area (Fig 4f), the allocated linear firebreak segments in the problem 5 solutions still tended to be evenly spaced across the region. Compared to the CERP edge removal solutions in problems 3–5, the CNDP node removal solutions in problems 1–2 allocated firebreaks in shorter segments and selected a few isolated nodes (Fig 7). Overall, the CERP problem 3 and 4 solutions achieved the most complete compartmentalization. The solutions to problem 6 that minimized the patch-based fire hazard measures could not effectively delineate compartments (Fig 7). While small portions of the removed nodes were allocated in 3- or 4-node segments, most of the treatments were allocated as a mix of clusters and single isolated nodes (Table 3). This indicates that using patch-based fire hazard measures will always require additional constraints to enforce the linear contiguity of the allocated firebreaks.

**Fig 8:**
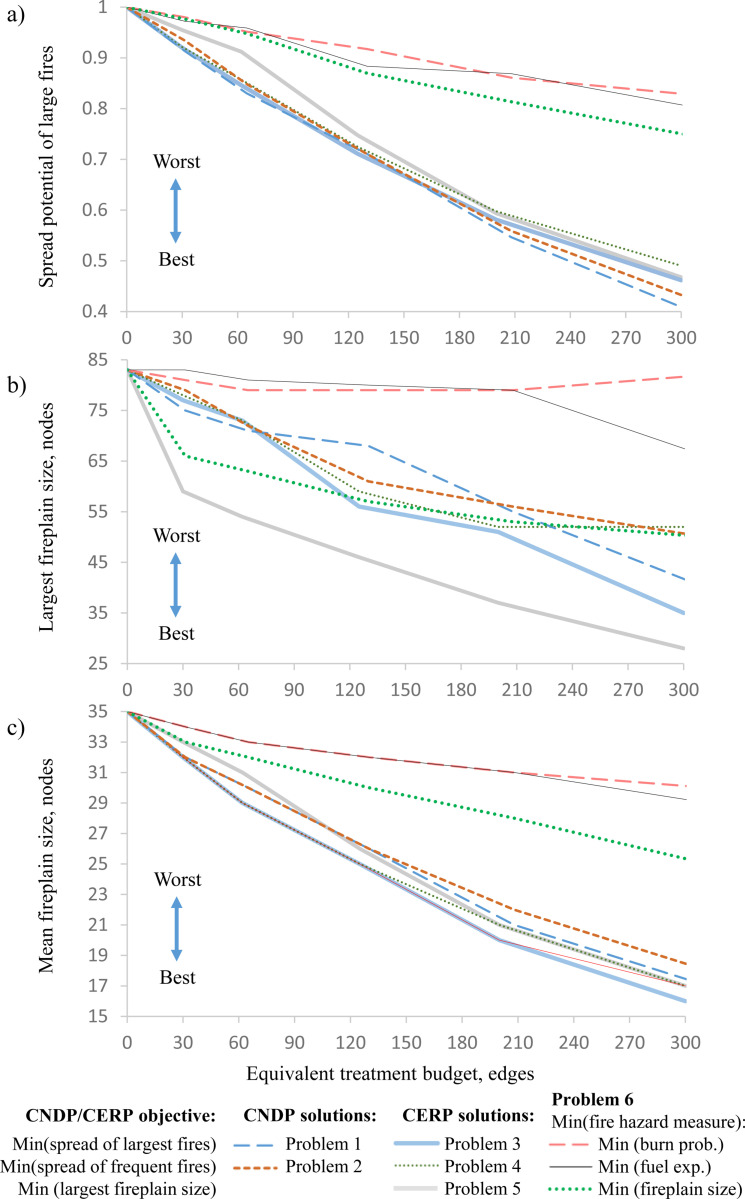
Objective value vs. the treatment budget. X-axis denotes the treatment budget level in the equivalent number of treated edges. The removed node budgets in the problem 1, 2 and 6 solutions were converted to the equivalent numbers of removed edges using a 2.5:1 cost ratio: a) spread potential of large fires; b) the largest fireplain size (characterizes the worst-case outcome of the spread of large fires); c) mean fireplain size (characterizes the average reduction of the potential fire spread area). Lower values on Y-axis indicate better outcomes.

**Fig 9:**
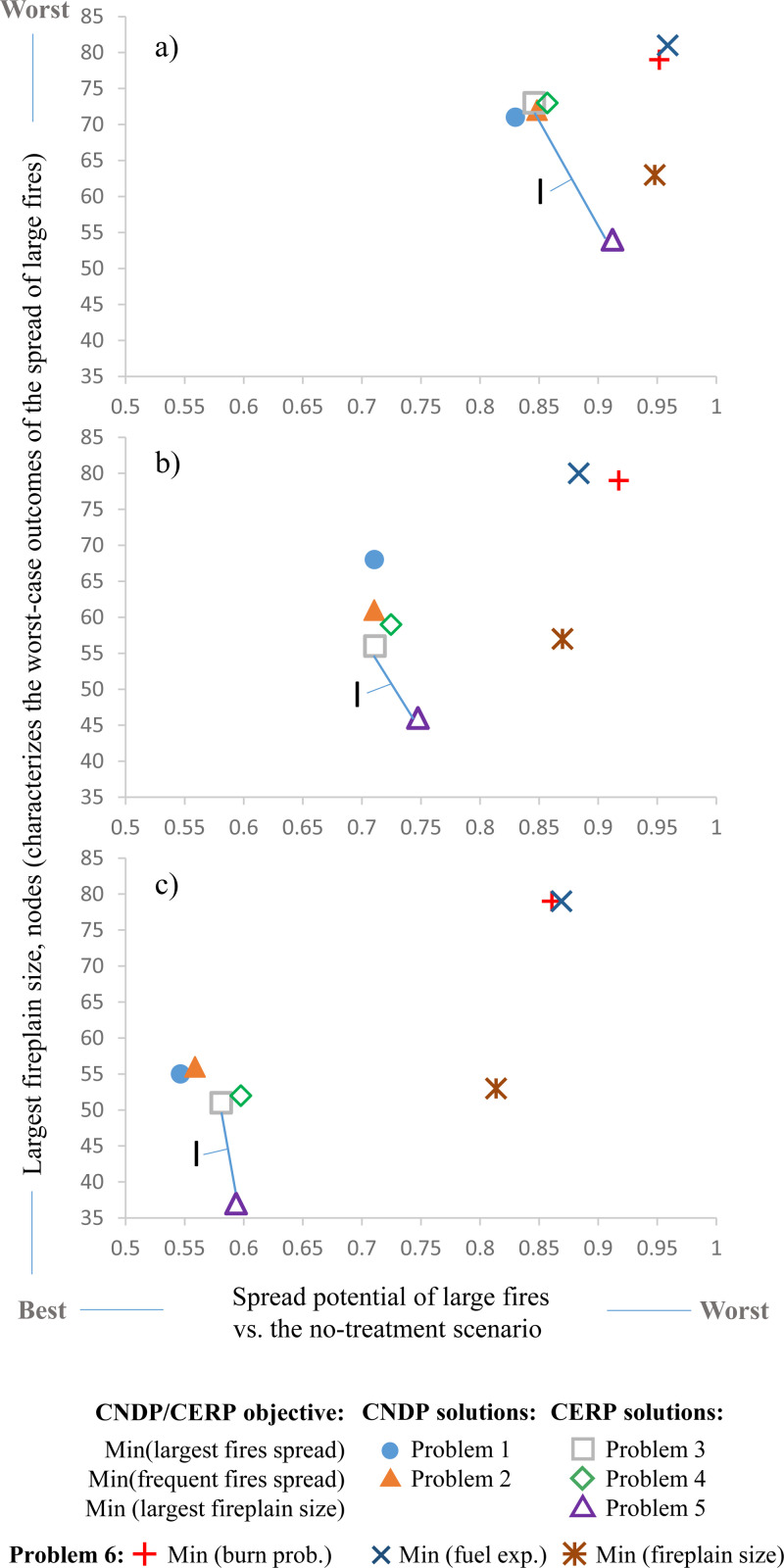
The trade-off between the capacity to reduce the spread potential of large fires vs. the reduction of the largest fireplain size. The treatment budget level: a) 25 nodes/ 62 edges; b) 50 nodes/ 125 edges; c) 80 nodes/ 200 edges. Callout I shows the trade-off between the problem 3 solution that minimizes the spread potential of large fires and the problem 5 solution that minimizes the largest fireplain size.

As the treatment budget increased, the solutions to problems 1–4 created more compartments (Fig 7). However, the reduction of wildfire spread risk yielded diminishing returns (Fig 8): the largest marginal reduction of fire spread risk was achieved at smaller budget levels. Edge removal problems 3–4 and node removal problems 1–2 achieved the most effective reduction of the fire spread risk across all budget levels (Fig 8a). Problem 5 solutions demonstrated the most effective reduction of the fireplain size (Fig 8b, Table 3). Notably, all CNDP and CERP solutions were able to effectively reduce both the average fireplain size (Fig 8c) and overall fire spread potential (Fig 8a). The trade-off between reducing the fireplain size or the fire spread potential across the landscape was small (Fig 9 callout I). This is because minimizing the number of node pairs with possible fire spread paths also reduced the number of long fire spread paths (which in turn reduced the sizes of the largest fireplains). By comparison, the problem 6 solutions that minimized the patch-based fire hazard metrics did not reduce the fire spread potential as effectively as the CERP or CNDP solutions, nor did the problem 6 solutions that minimized the precomputed fireplain size, despite some success with that specific objective (Fig 9).

### 3.3. Node removal vs. edge removal strategies

We compared the impacts of the CNDP and CERP-based firebreak allocation strategies using the node/edge removal cost ratio 2.5:1. Adopting this ratio, the reductions in fire spread risk and mean fireplain size among the problem 1–5 solutions appeared comparable (Fig 8a, 8c). However, by using this ratio, we likely overestimated the efficacy of node removal solutions because, on average, the removal of one node in these solutions eliminated five edges connecting that node to adjacent nodes (i.e., a 5:1 ratio). Since our intention was to assess the cost of creating linear firebreaks to define the compartments, we felt that our choice of a 2.5:1 ratio was reasonable. Whereas removal of a node in the CNDP solutions also removes all edges connected to that node, some of the removed edges may not have served as effective fire spread conduits. In contrast, removal of individual edges in the CERP solutions can be more nuanced and target only those edges critical for fire spread, which leads to a more efficient utilization of the fuel treatment budget. Overall, the CERP model produced stronger formulations with faster solving times than the CNDP even though the CERP model, on average, was 2.3 times larger in size than the CNDP.

## 4. Discussion

Recent catastrophic wildfires in Alberta, such as the fires in Slave Lake in 2011, Fort McMurray in 2016, and Jasper in 2024, have highlighted the need to accelerate the planning and implementation of fire risk-mitigation strategies. While national programs like FireSmart in Canada have encouraged many communities to undertake fuels modification, these efforts remained limited, as is the current level of prescribed and traditional burning [99,100]. Given the already-high level of land use in the region – mainly through forestry, mining and agriculture – there are numerous opportunities to integrate risk-mitigation strategies into regional land management (*cf*. [101]), particularly in high-use areas with substantial human infrastructure, as illustrated in a study that simulated wildfire behavior in east-central Alberta [102]. While there has been de facto implementation of compartmentalization ideas in some landscape-management plans in western Canada, somewhat analogous, in concept, to Potential Wildfire Operational Delineations (PODs) in the USA [103], full practical implementation could be challenging because it would need to include multiple land uses, land tenures, and economic actors. However, if the integration of land-use activities in the study region is borne out, we expect a rapid uptake of new fuels-reduction strategies. The proposed CERP approach, thanks to its capacity to track both the key fire spread paths and the potential burn area (the fireplain size), can assist with the development of such fire mitigation projects. Furthermore, the methodology can utilize model-based projections of current and future fire regimes, which helps capture the complexities of fire behavior in heterogeneous landscapes as well as the multitude of factors contributing to fire ignitions and spread.

The compartmentalization approach in our CERP formulations has advanced our previous work that applied critical node detection for optimizing fuel treatments in the following ways. The CERP addressed the chief limitation of the CNDP: the need to keep the node size compatible with the practical scale of the proposed fuel treatments. Furthermore, both node and edge removal formulations were able to track the presence-absence of connecting fire spread paths between node pairs, which helped account for factors controlling directional spread of fires. The practical benefits of this aspect were illustrated by our study region, which was characterized by prevailing winds from the southwest and was segmented into compartments by firebreaks that ran perpendicular to these winds. The CNDP and CERP problems did not always delineate full compartments. As shown in a previous study [50], effective reduction of fire spread risk may involve removal of isolated single nodes with high ignition risk. The compartments can be partial if they effectively intersect major fire spread paths and only need to cover the key locations where fires are likely to spread [104].

When presenting the CERP concept, we did not account for spatial variation of node (or edge) removal costs. This aspect would be important in remote landscapes with limited or no road infrastructure. Since our study area had reasonable road access established by decades of resource extraction activities, this simplification felt reasonable. However, the estimation of site access costs could become more complex if the fuel treatments had to be combined with other activities, such as targeted commercial harvesting of forest stands. In this situation, one would need to estimate where the benefits from commercial harvest offset the cost of treating the designated firebreak sites. Notably, integrating forest harvest planning into fuel treatment design not only reduces the potential loss of timber values, but also increases the feasibility of implementing and maintaining long-term wildfire mitigation strategies such as landscape compartmentalization.

Our results also pointed to important differences between the firebreak solutions based on patch-based fire hazard measures and those of the CERP. The key differences between the firebreak allocation strategies in the examined models stemmed from the nature of the performance metric in the objective function and the associated wildfire information used to guide the allocation decisions. The CNDP and CERP models incorporated fire information in the form of a two-dimensional matrix of fire spread likelihoods *p*_*ij*_ (or binary indicators *q*_*ij*_) between all node pairs in the area, which enabled tracking of the impact of firebreaks on all directional fire spread vectors. As the treatment budget level increased, these models created a system of compartments that encapsulated almost all major hotspots with high burn likelihoods (Fig 7). By comparison, fire hazard information at the level of a single-dimensional patch metric in problem 6 was insufficient to characterize the potential fire spread directions and likely spread distances in the model. To work effectively, problem 6 would require additional constraints to enforce linearity of the firebreaks, like the contiguity constraints in [48] and [50], but this would not guarantee effective interdiction of the key fire spread paths.

The CERP problem solutions elucidated the importance of the uncertainty assumptions in estimating the fireplain configurations. In our study, we delineated the fireplains Ω_*i*_ around nodes *i* as unions of the footprints of the fires ignited in *i* (as predicted by the fire growth model). This was a depiction of the worst-case fire spread outcomes because the fireplain accounted for the maximum possible fire spread area from node *i* and the fireplain size was shaped by the occurrence of large fires. Since large fires are rare, this strategy may be less effective when the fire behavior is dominated by small fires. Adding the fire spread probability parameter *p*_*ij*_ to the objective equation in problem 4 helped address this issue. We acknowledge that other prioritization schemes can be applied to adjust the priority of decision variables *u*_*ij*_; for example, the fire spread distance *ij* or the fire intensity along the fire spread path.

One potential hurdle in the practical adoption of the methodology is the need to assemble complex fire weather and fuel composition datasets for undertaking fire behavior simulations with the fire growth model. Also, the combinatorial complexity of both CNDP and CERP may limit their applications to moderate-size fuel networks. Applying them to the data of large landscapes may require restricting the scope of model decision variables, either by adding constraints that narrow the range of suitable site access conditions or imposing stricter site selection criteria for the firebreak sites. Another potential workaround to reduce the problem size is to coarsen the spatial resolution of the fuel network selectively in the areas where the establishment of firebreaks is unlikely or in remote locations outside the main fire spread corridors.

Potential extensions of the CERP formulation could include applications to find the configuration of firebreaks to protect critical human infrastructure in wildland-urban interface areas [105–107]. For example, the formulation can be modified following the approach in [39] for the protection of industrial infrastructure from wildfires. Alternatively, the CERP can be incorporated into a blended approach that combines the interdiction of critical fire spread corridors with targeted treatments of potential fire ignition hotspots and locations with high-value infrastructure assets.

Our model did not track the effectiveness of firebreak segments. Firebreak effectiveness is correlated with local burn intensity; however, the overall capacity to stop fire spread may be compromised by long-range ember transport (i.e., fire spotting), which would require increasing the level of fuel treatment across all allocated segments to make the firebreaks effective. The 2.15-km cross-sectional width of fuel treatments in this study should prevent most possibilities of spotting in the area’s forest types [87,88] and limit fire spread under a wide range of weather conditions. Nevertheless, these treatments should never be considered infallible. Extreme weather may, on occasion, overcome limitations imposed by fuels and enable fires to spread through vegetation considered to have low flammability [108,109], albeit at a comparatively lower intensity [110]. Notably, fuel treatments are rarely designed to function independently of fire management activities. They are usually undertaken in conjunction with tactical operations, such as strip burning or back burning, where they provide anchor points for suppression teams, and are augmented by further fuel removal, thereby increasing their effectiveness [59,111].

Our model could be modified to track firebreak effectiveness as follows: for each simulated fire footprint with the fire growth model, fire intensity values would be recorded at each location within that footprint and compose a fire propagation graph that depicts the possible fire spread (including the fire intensities) from the ignition location towards the fire perimeter (e.g., using the approach presented in [112]). Then, the optimization model would need to track the impacts of firebreak allocation through all simulated fire propagation graphs. For each fire propagation graph, firebreak failures could be assessed by comparing the fire intensities in the firebreak locations against the fire intensity threshold that a given firebreak width (or level of fuel treatment) is estimated to contain. Such a model would consider the trade-off between the selection of the firebreak width (or level of treatment) with the corresponding acceptable failure level and the overall length of the firebreak system in the landscape. In this context, the firebreak allocation problem could be formulated analogously to a financial portfolio problem that evaluates the trade-off between the likelihoods of firebreak failures (analogous to asset volatility) and the mean fire hazard reduction (analogous to the portfolio return).

## Supporting information

S1 Appendix Burn-P3 fire growth model simulations. (DOC)

S2 Appendix Network node-based metrics. (XLSX)
