# Supplementary material for: Evaluating fuelbreak strategies for compartmentalizing a fire-prone forest landscape in Alberta, Canada
Source: PLoS One. 2025 May 21;20(5):e0321722. doi: 10.1371/journal.pone.0321722 (PMC12094731; doi:10.1371/journal.pone.0321722)
Supplement: S1 Appendix — (DOC) [file pone.0321722.s001.doc]

**APPENDIX S1. BURN-P3 FIRE GROWTH MODEL SIMULATIONS**

We simulated fires across our study area using the Burn-P3 model (Parisien et al. 2005). Burn-P3 simulates forest fires across a landscape using the Prometheus fire growth engine (Tymstra et al. 2010). The Prometheus algorithm is based on the Canadian Forest Fire Behavior Prediction (FBP) System (Stocks et al., 1989). Burn-P3 repeatedly generates the stochastic realizations of a “burn year” (also termed the model replications). The ignition locations and daily fire weather conditions are based on randomized draws from historical probability distributions of the key weather parameters. For each replication of a burn year, Burn-P3 generates the spatial pattern of ignition locations and simulates the growth of ignited fires according to weather conditions, fuel composition and topography. The Prometheus algorithm simulates the fire intensity (in kW/m), linear rate of fire spread, fuel consumption and other metrics which characterize wildfire behavior. The model uses multiple input datasets such as spatial distributions of fuels, ignition likelihoods, elevation and daily weather parameters (see Table S1.1). For each replication of a burn year, for each day of an active fire season, Burn-P3 draws randomly the distribution of fire weather conditions, and the parameters which depict the possible fire spread durations and the time spans of an active fire burn during the day. The model also uses historical fire records to select the number of ignitions per iteration, cause and location via randomized draws (Parisien et al. 2005).

The raw Burn-P3 outputs used in our analyses included approximately 32000 simulated fire footprints with the footprints extending over more than one hexagon and the corresponding ignition locations based on the simulations of 60000 replications of a burn year. We have also summarized these outputs into burn probabilities, which estimated the relative likelihoods of fire occurrence at a given location (Parisien et al. 2019). The fire simulations were carried out at 100-meter spatial resolution. We have set the minimum fire size to 3 ha. For the study area, we prepared all spatial inputs including a 20-km additional buffer around the study area to remove edge effects.

We derived the maps of fuel types and other spatial inputs from the datasets provided by Weyerhaeuser Inc. and the Canadian Forest Service. The map of fuel types followed the fuel classification from the Canadian Fire behavior Prediction System (Stocks et al. 1989). Burn-P3 modifies fuels based on seasons, which determines the presence of foliage on deciduous trees and whether grasses are ‘cured’. Grass fuel was set to cure at 60%.

The model also required spatial data on elevation, fuels, relative probabilities of ignitions by season, wind directions and speed (Table S1.1). Spatial ignition patterns were based on historical fire records from 2017 to 2019 collected at the Alberta government weather stations in the study area. All spatial inputs were composed at a 100-m resolution. Other non-spatial inputs included the frequency distributions of escaped fires larger than 1 ha, the number of spread days per fire. The daily fire weather and associated Fire Weather Index parameters were grouped by season according to Van Wagner (1987). The model also required defining the start and end of the spring and summer periods and fuel green-up dates. We assumed that spring fire weather occurs in the study area from April 15 to June 10, and summer weather occurs after June 10.

The number of spread days per fire was drawn from the records of historic fires in the surrounding regions from the Canadian National Fire Database (NRCan 2017). Following the previous fire behavior simulation study in northeastern Alberta (Stockdale et al., 2019), we set per-day burning durations to six hours. The probability of ignitions was stratified spatially by the density of historic lighting strikes (ECCC 2023) and the proximity to roads based on the road map layer provided by Weyerhaeuser Inc. We described the topography of the study area using Canadian Digital Elevation Model (NRCan 2015) and used the WindNinja program (https://www.firelab.org/project/windninja) to calculate the impacts of topography on wind directions and speeds for each of the eight-cardinal directions.

**Table S1.1. Burn-P3 input variables.**

| Model input | | Data type | Description |
| --- | --- | --- | --- |
| *Static* | |  |  |
|  | Topography | Continuous raster | Elevation (m) |
|  | Topographical wind speed and wind direction grids | Continuous raster (16 grids) | Influence of topography on wind direction and wind speed; produced for the eight cardinal directions |
|  | Fuels | Categorical raster | Canadian Fire Behavior Prediction System fuel and non-fuel types representing expected fire behavior; 15 total fuel types within the study area |
|  | Seasons | Setting | Dates defining the start and end of the spring period and summer period for selection of fire weather, green-up dates, and grass curing percentage. Spring weather occurs from April 15 to June 10, and summer weather occurs after June 10. |
|  | Minimum fire size | Setting | Minimum size at which fires are retained by Burn-P3. Set to 3 ha |
| *Stochastic* | |  |  |
|  | Ignition location grids | Continuous raster | Modeled probability of human and lightning ignition locations based on historical ignition data |
|  | Ignitions by season, fire zone, and cause of fires | Frequency distribution | Proportion of fire ignitions by season |
|  | Daily fire-weather | Numeric list | Daily weather in which fires would be expected to ignite and spread, based on the Fire Weather Index System (i.e., when the daily value of the FWI ≥ 13), partitioned by season and weather zone |
|  | Spread event days | Frequency distribution | Fire duration; derived from number of fire weather days in which fires have the potential to achieve significant spread in both current and projected weather |
|  | Hours of burning | Frequency distribution | Daily hours in which fires can spread; set to vary up to 6 hours |

**REFERENCES:**

Environment and Climate Change Canada (ECCC) 2023. Lightning Density Data. https://open.canada.ca/data/en/dataset/75dfb8cb-9efc-4c15-bcb5-7562f89517ce.

Forestry Canada Fire Danger Group. Development and structure of the Canadian Forest Fire Behavior Prediction System, Forestry Canada Information Report ST-X-3, Ottawa, ON. 1992.

Natural Resources Canada (NRCan). 2015. Canadian Digital Elevation Model, 1945-2011.

Natural Resources Canada (NRCan). 2017. Canadian National Fire Database – Agency Fire Data. Natural Resources Canada, Northern Forestry Centre, Edmonton, Alberta. http://cwfis.cfs.nrcan.gc.ca/en_CA/nfdb.

Parisien MA, Kafka V, Hirsch KG, Todd JB, Lavoie SG, Maczek PD. Mapping Wildfire Susceptibility with the BURN-P3 Simulation Model; Natural Resources Canada, Canadian Forest Service, Northern Forestry Centre, Information Report NOR-X-405: Edmonton, AB, 2005.

Parisien MA, Dawe DA, Miller C, Stockdale CA, Armitage OB. Applications of simulation-based burn probability modelling: a review. International Journal of Wildland Fire 2019; 28: 913-926.

Stockdale C, Barber Q, Saxena, A., Parisien M-A. Examining management scenarios to mitigate wildfire hazard to caribou conservation projects using burn probability modeling. Journal of Environmental Management 2019; 233: 238-248.

Stocks BJ, Lynham TJ, Lawson BD, Alexander ME, Wagner CEV, McAlpine RS, Dubé DE. Canadian Forest Fire Danger Rating System: An Overview. For. Chron. 1989; 65: 258-265.

Tymstra C, Bryce RW, Wotton BM, Taylor SW, Armitage OB. Development and Structure of Prometheus: The Canadian Wildland Fire Growth Simulation Model; Natural Resources Canada, Canadian Forest Service, Northern Forestry Centre, Information Report NOR-X-417: Edmonton, AB, 2010.

Van Wagner CE. Development and structure of the Canadian Forest Fire Weather Index System. Canadian Forestry Service, Environment Canada Forestry Technical Report 35; Ottawa, ON, 1987.
